# Management of Bilateral Diffuse Uveal Melanocytic Proliferation (BDUMP)—A Case Report

**DOI:** 10.3390/medicina59122158

**Published:** 2023-12-13

**Authors:** Nataša Drača, Emma Grace Orešković, Ratimir Lazić, Marija Vukojević, Andrea Radolović Bertetić, Nenad Vukojević

**Affiliations:** 1 Svjetlost Eye Clinic and University Hospital, 10000 Zagreb, Croatia; emma.oreskovic@svjetlost.hr (E.G.O.); ratimir.lazic@svjetlost.hr (R.L.); 2 Department of Ophthalmology, General Hospital Pula, 52100 Pula, Croatia; mvukojevic97@gmail.com; 3 Institute of Emergency Medicine of Sisak-Moslavina County, 44000 Sisak, Croatia; 4 Department of Opthalmology, University Hospital Rebro, 10000 Zagreb, Croatia; andrea.radolovic@gmail.com (A.R.B.); nvukojev@gmail.com (N.V.)

**Keywords:** cataract, BDUMP, ovarian cancer, paraneoplastic syndrome

## Abstract

*Background and Objectives*: This study reports a case of a 62-year-old patient experiencing a significant decline in vision over the past three months. The initial best-corrected visual acuity (BCVA) of 20/20 in both eyes diminished to 20/200 in the right eye (RE) and counting fingers (CF) in the left eye (LE) within this timeframe. The patient was diagnosed with stage 4 ovarian cancer just one month before the significant vision deterioration. *Materials and Methods*: A thorough ophthalmologic examination revealed a notable progression of cataracts and the presence of subretinal fluid on the posterior pole, accompanied by choroidal thickening. The right eye exhibited multifocal, orange-pigmented, and elevated choroidal lesions, while the left eye’s fundus examination was impeded by dense cataracts. Optical coherence tomography (OCT) revealed bilateral choroidal thickening with overlying folds and subretinal fluid, and ultrasound imaging of the choroidal lesions indicated moderate homogenous internal reflectivity. *Results*: The patient received a diagnosis of BDUMP (bilateral diffuse uveal melanocytic proliferation), a paraneoplastic syndrome marked by simultaneous, bilateral, painless vision loss and the rapid onset of bilateral cataracts with serous retinal detachments. Despite cataract extraction, the expected visual recovery was not achieved (RE: CF; LE: 2/200, respectively). Plasmapheresis showed some success in stabilizing vision loss attributed to serous retinal detachments. *Conclusions*: BDUMP necessitates addressing the underlying malignancy for effective treatment. Left untreated, it can lead to near blindness within a year. The prognosis remains grim, with an average survival time ranging from 12 to 15.7 months from the time of diagnosis. Considering this case report, it is crucial to establish effective management plans and further investigate potential treatment methods and predictive markers centered around BDUMP. Collaboration between healthcare professionals and researchers is crucial in addressing the complexities of BDUMP, as the timely diagnosis and treatment of the disease remains a top priority.

## 1. Introduction

Bilateral diffuse uveal melanocytic proliferation (BDUMP) is a relatively rare paraneoplastic syndrome impacting the eyes. Originating from Machemer’s initial description in 1966 [1], its comprehensive characterization was later undertaken by Gass and colleagues in 1990 [2]. BDUMP manifests through a distinctive set of features: discreet patches in the posterior fundus, early hyper-fluorescent multifocal regions, pigmented or non-pigmented uveal melanocytic tumors with a slight elevation and thickening of the uveal tract, exudative retinal detachment, and a swift progression of cataracts. Gass’s “leopard spots”, characterizing the reticulated ocular fundus pattern, are colloquially known as the “gyrate pattern”. While the reticulated depigmentation pattern is characteristic of BDUMP, its diagnostic specificity is tempered by its occurrence in other bilateral conditions such as metastatic cancer, leukemic choroidal infiltration, and lymphoma, all of which can manifest bilaterally [3].

Exceptionally rare cases of BDUMP may exhibit multiple pigmented nodules devoid of the classic “leopard spots”. This comprehensive exploration, shedding light on BDUMP’s ocular intricacies, underscores the necessity of nuanced diagnostic discernment and therapeutic approaches for optimal clinical management. It is imperative to acknowledge the diversity of its presentation and to urge clinicians towards a meticulous understanding for the purpose of accurate diagnosis and effective therapeutic intervention in this complex ocular syndrome. As clinicians encounter variations in BDUMP’s manifestation, the need for a refined comprehension becomes more apparent, emphasizing the intricate nature of this syndrome and the importance of individualized patient care strategies.

The term “BDUMP” was initially coined by Barr and colleagues [4] to describe a spectrum of pathologies. Their delineation highlighted four crucial ocular clinical features associated with this syndrome: bilateral involvement of the uveal tracts in a diffuse manner; a predominantly benign cytological composition characterizing melanocytic lesions; the absence of evidence indicating metastasis from the melanocytic tumors; and confirmation of a linked systemic malignancy, substantiated through biopsy.

The pathogenesis underlying melanocytic infiltration in BDUMP remains enigmatic. It is theorized that either a substance secreted by the tumor or an antibody stimulated by the tumor triggers the benign proliferation of choroidal melanocytes [5]. Extensive discussions on the pathologic correlates of BDUMP have been documented in nearly 100 cases [3], underscoring the ongoing efforts to unravel its intricacies.

Typically diagnosed in individuals during their sixth to seventh decade, BDUMP predominantly affects those previously diagnosed with diverse non-ocular cancers. Noteworthy patterns reveal a higher frequency of female urogenital cancers (69%) and male lung carcinomas (52%). Sporadic cases encompass cancers of the pancreas, esophagus, breast, liver, Bartholin gland, renal cells, and central nervous system lymphoma [3].

Following BDUMP diagnosis, the average survival period hovers slightly over 15 months, with a range from 10 months to 5 years [6]. Unfortunately, the visual prognosis for BDUMP is bleak, marked by progressive visual deterioration stemming from dense bilateral cataract formation and chronic subretinal fluid accumulation, leading to secondary degeneration of RPE cells and photoreceptors. The prognosis is notably graver in cases involving macular detachment [1,2] due to the critical role the macula plays in central vision. Macular detachment can lead to severe impairment or a loss of central vision, exacerbating the already-challenging visual prognosis associated with BDUMP. The impact on macular function intensifies the overall severity of visual impairment in these cases, posing additional challenges regarding effective management and rehabilitation.

In a noteworthy case by Duong et al., a patient with BDUMP exhibited exceptional resilience, surviving an additional 10.5 years post-bilateral enucleation [7].

In this case report, we present a unique case of a 62-year-old female patient diagnosed with BDUMP just one month after a clear cell adenocarcinoma diagnosis in the right ovary. This case contributes to the evolving understanding of BDUMP’s complex interplay with systemic malignancies, offering valuable insights into its diagnostic challenges and therapeutic considerations.

## 2. Materials and Methods

**Figure 1:**
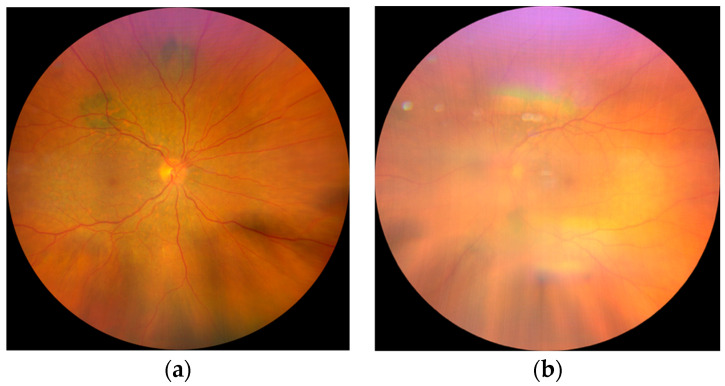
Fundus photography: (**a**) Fundus of the RE shows diffuse, multifocal, pigmented lesions around the arcades and mild edema of the posterior pole; (**b**) barely visible fundus of the LE due to the cataract.

The patient’s swift referral to our clinic was prompted by the alarming progression of bilateral cataracts over a span of merely three months. A precipitous decline in the best-corrected visual acuity (BCVA) from 20/20 to 20/200 in the right eye (RE) and counting fingers (CF) in the left eye (LE) prompted a comprehensive examination. Noteworthy findings included Tyndall + cells in the anterior chamber; dense bilateral cataracts with greater prominence in the LE; and multifocal lesions on fundus examination, particularly discernible at orange pigment locations, though hindered by LE cataract opacity (Figure 1).

**Figure 2:**
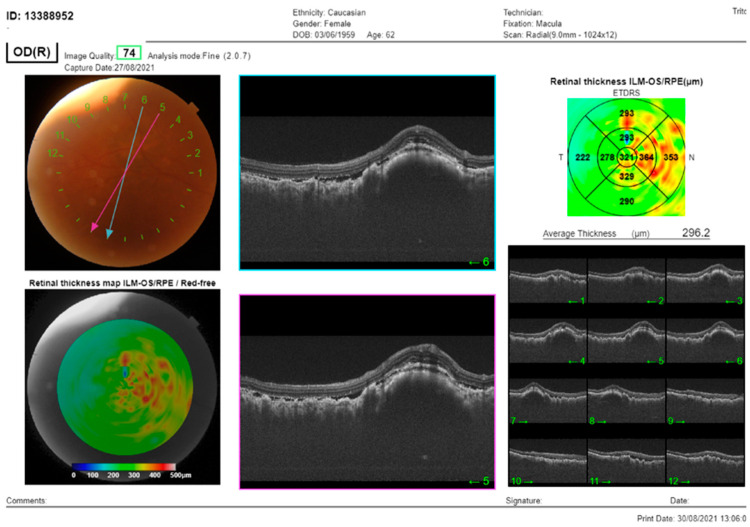
The SS-OCT (DRI OCT Triton, Topcon Co, Tokyo, Japan) showed choroidal thickening with overlying folds and subretinal fluid. OCT of the macula of the RE: cross-section through the lesion showed thickening of the choroid, with subretinal fluid and overlying folds of RPE.

In the right eye, the OCT signal was notably weakened by the presence of dense cataracts. Nevertheless, despite this impediment, subretinal fluid and choroidal thickening were both still evident in this eye (Figure 2).

**Figure 3:**
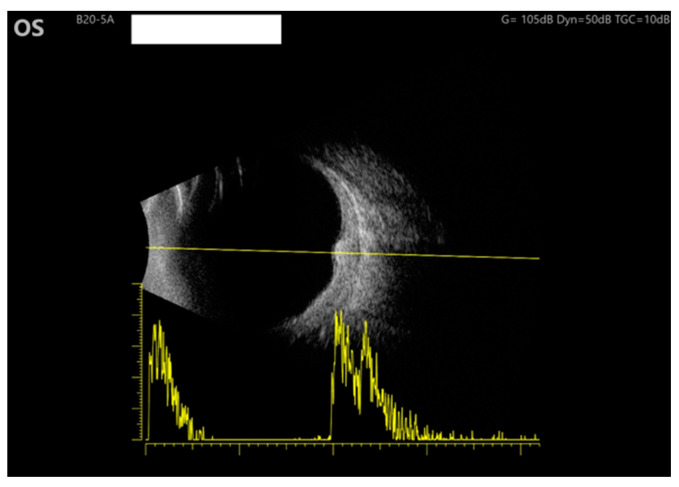
Ultrasound of the LE: B scan demonstrates moderate internal reflectivity through the lesion and diffuse choroidal thickening.

An ultrasound was performed on the left eye, revealing moderate internal reflectivity within the lesion and diffused thickening of the choroid. The B-scan provided detailed imaging, allowing for a thorough evaluation of the area (Figure 3).

Given the patient’s history of ovarian cancer, hysterectomy, and bilateral adnexectomy due to right ovarian adenocarcinoma, a presumptive diagnosis of bilateral diffuse uveal melanocytic proliferation (BDUMP) was established.

Post-surgery, chemotherapy initiation was truncated after three cycles due to severe toxic sensorimotor polyneuropathy. Concurrently, chronic renal insufficiency (Stage IV), secondary anemia, hypoalbuminemia, and hypocalcemia were identified as coexisting conditions. Despite cataract surgery on both eyes, the patient’s vision deteriorated, which was attributed to chronic serous retinal detachment (RE: CF, LE: 30/200).

Efforts to address ocular complications included plasmapheresis, which, despite implementation, yielded no substantial changes in visual acuity or posterior pole lesions. Aflibercept (Eylea) application minimally impacted the subretinal fluid. Immunomodulatory therapy, though considered, was precluded by renal insufficiency and elevated liver enzymes. Subsequently, subretinal fibrosis emerged, progressing with nasal retinal elevation in the right eye, and this prompted ongoing observation for potential vitrectomy. This detailed chronicle underscores the intricate interplay of ocular pathology, systemic conditions, and therapeutic interventions in the management of a complex case, emphasizing the need for nuanced approaches and ongoing vigilance.

## 3. Results

With treatment initiated in 2021, the patient is currently undergoing a comprehensive therapeutic regimen. Remission was achieved following a series of interventions, including ovariectomy, hysterectomy, and curative chemotherapy, which had to be discontinued due to neurological complications. Despite these systemic efforts, the patient experienced a significant decline in vision, reaching counting fingers (CF) on the right eye (RE) and 2/200 in the left eye (LE). Notably, various ocular interventions were implemented, encompassing cataract surgery, plasmapheresis, intravitreal anti-VEGF (aflibercept) administration, and systemic and local corticosteroid therapy.

Immunomodulatory therapy, a potential avenue for intervention, was precluded due to the emergence of renal and liver complications. Although inflammation in the anterior segment abated, the persistence of subretinal fluid and lesions precipitated the development of subretinal fibrosis, raising concerns regarding the potential for tractional retinal detachment. Consequently, the consideration of vitrectomy on the RE has become imperative. This case accentuates the intricate balance required in managing both systemic and ocular complications, underscoring the necessity for ongoing research to refine therapeutic strategies for optimal patient outcomes.

## 4. Discussion

Bilateral diffuse uveal melanocytic proliferation (BDUMP) poses a unique challenge in its management, necessitating a comprehensive approach to address both the primary malignancy and associated ocular manifestations. The initial focus of treatment revolves around tackling the primary malignancy through surgical removal, radiation therapy, or systemic chemotherapy [4]. This dual-pronged strategy aims not only to curb the progression of the primary malignancy, but also to alleviate the ocular symptoms linked to BDUMP.

A pivotal aspect of BDUMP pathogenesis lies in systemic factors triggered by the primary malignancy, potentially expediting the progression of ocular symptoms. Miles and colleagues shed light on the presence of a stimulatory factor in the serum and plasma of BDUMP patients, termed the “CMEP factor” due to its role in fostering the elongation and proliferation of cultured melanocytes [8]. While plasmapheresis emerges as a potential avenue to eliminate plasma CMEP, its efficacy appears to exhibit variability among cases. In our specific case, plasmapheresis did not yield a significant improvement in clinical outcomes, underscoring the intricate and individualized nature of BDUMP treatment responses.

Notable differential ocular pathologies must also be ruled out when efficaciously diagnosing BDUMP. For example, five instances suggestive of UDUMP have been reported in the literature, but none underwent histological verification [3]. The available clinical follow-up, averaging less than 6 months, is limited, raising concerns about the potential omission of sequential or asymmetric ocular involvement, a known occurrence in the development of BDUMP. Notably, one case documented simultaneous unilateral intraocular lymphoma and UDUMP without histopathologic examination [8,9]. Therefore, caution is advised when interpreting cases of UDUMP, and further scrutiny is crucial, particularly in the context of BDUMP [3,10].

Moreover, it is crucial to discern BDUMP from other conditions presenting with similar ocular manifestations. Choroidal melanoma warrants differentiation due to its potentially malignant nature and distinct therapeutic approaches [10]. Skin melanoma metastases to the choroid pose are also on the differential, emphasizing the importance of distinguishing between primary ocular manifestations and secondary malignancies [11,12]. Next, choroidal nevi associated with neurofibromatosis 1 introduced a differential diagnosis with a genetic component, necessitating careful examination to elucidate the underlying etiology and whether it was genetic or environmental [12]. Lastly, multiple congenital hypertrophy of the retinal pigment epithelium (CHRPE) lesions associated with Gardner syndrome must also be considered, highlighting the significance of recognizing syndromic implications [12]. A comprehensive list of differential diagnoses not only facilitates accurate disease identification and classification, but also serves as a guiding framework for the development of appropriate and tailored patient management strategies and demonstrates the critical necessity of precise diagnostic discernment within the intricate landscape of BDUMP, acknowledging the nuances and potential complexities that may arise during the diagnostic process.

The exploration of intravitreal anti-VEGF agents has shown promise in certain instances by mitigating intra-retinal fluid accumulation [4]. In our pursuit of effective interventions, intravitreal aflibercept application demonstrated a discrete reduction in subretinal fluid; however, this did not translate into improved visual acuity, emphasizing the complexity of managing BDUMP. Additionally, traditional approaches, including ocular radiation, sub-retinal fluid drainage, and corticosteroids, have generally exhibited limited efficacy [9,10].

To address the inflammatory component of BDUMP, systemic corticosteroids were administered in a tapered-dose fashion, complemented by local application of both non-steroid and steroid anti-inflammatory drops. This multifaceted approach reflects the intricate interplay between immunological responses and ocular manifestations in BDUMP. Notably, the consideration of immunomodulatory therapy was contemplated, but ultimately deferred due to the presence of renal and liver complications in our patient. This decision aligns with the existing literature highlighting the potential for BDUMP as a complication of immune-modulating therapy [11].

The treatment of BDUMP requires personalized strategies that consider both ocular and systemic aspects of the condition. Continued research and clinical exploration are imperative to unraveling the intricacies of BDUMP pathogenesis and refining therapeutic approaches for enhanced patient outcomes. As we navigate the complexities of this rare ocular disorder, the integration of evolving insights into clinical practice remains paramount.

## 5. Conclusions

Ultimately, there is a compelling need for further investigation to identify potential primary malignancies associated with BDUMP. Despite its rarity, with an incidence of approximately 4.4 cases per year in 2017 [3], BDUMP demands personalized management strategies. The symptoms, notably the swift onset of cataracts coupled with advanced vision loss, should be recognized as crucial warning signs for identifying this syndrome, particularly when accompanied by anamnestic evidence of an underlying primary malignancy. Conducting thorough systemic examinations in patients suspected of having BDUMP is of paramount importance to pinpoint potential primary malignancies and establish a definitive link between the malignancy and ocular changes. Such an approach facilitates early treatment initiation, leading to improved prognoses, a critical consideration given the extremely poor prognosis associated with BDUMP. Understanding that the slow spread of the primary malignancy can potentially extend the survival time from the mean of 15.6 months to exceptional cases ranging from 4 to 9 years underscores the significance of early detection and intervention [11]. This comprehensive approach not only aids in refining management strategies, but also holds promise in enhancing patient outcomes and survival rates in the context of BDUMP.

## Data Availability

Data is contained within the article. The data presented in this study are available in the reference section of this case report.
